# Molecular basis for the recruitment of the Rab effector protein WDR44 by the GTPase Rab11

**DOI:** 10.1016/j.jbc.2022.102764

**Published:** 2022-12-01

**Authors:** Matthew C. Thibodeau, Noah J. Harris, Meredith L. Jenkins, Matthew A.H. Parson, John T. Evans, Mackenzie K. Scott, Alexandria L. Shaw, Daniel Pokorný, Thomas A. Leonard, John E. Burke

**Affiliations:** 1 Department of Biochemistry and Microbiology, University of Victoria, Victoria, British Columbia, Canada; 2 Department of Biochemistry and Molecular Biology, The University of British Columbia, Vancouver, British Columbia, Canada; 3 Max Perutz Labs, Department of Structural and Computational Biology, Vienna, Austria; 4 Department of Medical Biochemistry, Medical University of Vienna, Vienna, Austria

**Keywords:** Rab GTPases, Rab11, WDR44, HDX-MS, hydrogen exchange, AlphaFold, BLI, biolayer interferometry, bME, β-mercaptoethanol, D2O, deuterium oxide, FIP, family of Rab11-interacting protein, GFB, gel filtration buffer, GST, glutathione-*S*-transferase, H/D, hydrogen/deuterium, HDX-MS, hydrogen/deuterium exchange mass spectrometry, MS, mass spectrometry, Ni–NTA, nickel–nitrilotriacetic acid, PDB, Protein Data Bank, *PDK1*, phosphoinositide-dependent kinase 1, *PI4KB*, phosphatidylinositol 4 kinase III β, RBD, Rab-binding domain, TCEP, Tris(2-carboxyethyl)phosphine, TEV, tobacco etch virus, UPLC, ultraperformance liquid chromatography, *WDR44*, WD repeat–containing protein 44

## Abstract

The formation of complexes between Rab11 and its effectors regulates multiple aspects of membrane trafficking, including recycling and ciliogenesis. WD repeat–containing protein 44 (WDR44) is a structurally uncharacterized Rab11 effector that regulates ciliogenesis by competing with prociliogenesis factors for Rab11 binding. Here, we present a detailed biochemical and biophysical characterization of the WDR44–Rab11 complex and define specific residues mediating binding. Using AlphaFold2 modeling and hydrogen/deuterium exchange mass spectrometry, we generated a molecular model of the Rab11–WDR44 complex. The Rab11-binding domain of WDR44 interacts with switch I, switch II, and the interswitch region of Rab11. Extensive mutagenesis of evolutionarily conserved residues in WDR44 at the interface identified numerous complex-disrupting mutations. Using hydrogen/deuterium exchange mass spectrometry, we found that the dynamics of the WDR44–Rab11 interface are distinct from the Rab11 effector FIP3, with WDR44 forming a more extensive interface with the switch II helix of Rab11 compared with FIP3. The WDR44 interaction was specific to Rab11 over evolutionarily similar Rabs, with mutations defining the molecular basis of Rab11 specificity. Finally, WDR44 can be phosphorylated by Sgk3, with this leading to reorganization of the Rab11-binding surface on WDR44. Overall, our results provide molecular detail on how WDR44 interacts with Rab11 and how Rab11 can form distinct effector complexes that regulate membrane trafficking events.

Coordinated membrane trafficking of vesicular cargo to the correct intracellular locations is fundamental to complex eukaryotic life. A critical player in membrane trafficking are the Rab GTPases, a subfamily of the Ras superfamily of GTPases that play important roles in membrane identity and trafficking (1, 2, 3, 4). Rab GTPases regulate many steps of membrane trafficking by interacting with effector proteins along actin and tubulin networks (5). Rab GTPases act as molecular switches, alternating between a GTP-bound “on” state and a GDP-bound “off” state, with binding to effectors being dependent on conformational changes of the switch regions driven by nucleotide binding (2, 3). The Rab GTPase Rab11 is a master regulator of exocytic and recycling processes and critical in guiding proteins to the cell surface (5). Rab11 is a family of GTPases encompassing three unique genes Rab11a, Rab11b, and Rab11c (Rab25), sharing 89% sequence identity for Rab11a/Rab11b and 61% for Rab11a/Rab11c. Rab11a localizes to early endosomes, recycling endosomes, trans-Golgi network, and post-Golgi vesicles and is important in endosomal recycling and ciliogenesis (5, 6). Active GTP-bound Rab11 at intracellular membranes recruits an extensive set of effector proteins, including tethering factors, molecular motors, scaffolding proteins, and kinases to control vesicular trafficking events (3). Understanding the molecular basis of how unique effectors bind to Rab11 is essential in defining its various roles in fundamental membrane trafficking events.

Binding of Rab GTPases to their effectors is driven through nucleotide-dependant conformational changes in regions defined as switch I and switch II, with most effectors only binding to GTP-bound Rab (7, 8). The switches are canonically disordered when Rab is bound to GDP, with GTP binding stabilizing both switch I and II, which increases the affinity for effectors (3). Multiple different Rab11 effectors have been identified, including the mammalian family of Rab11-interacting proteins (FIPs) (9). FIPs share a homologous C-terminal coiled-coil Rab11-binding domain (RBD), where interaction with Rab11 drives FIP homodimerization resulting in heterotetrameric (2:2) complexes (10, 11, 12). Rab11 also interacts with the C-terminal globular tail domain of myosin V^13^ and the exocyst subunit Sec15 (14) in a GTP-dependent manner through different interfaces compared with FIP family proteins. In addition to these canonical interactions, Rab11 is unique in that it can bind to effectors through an interface outside the switch regions, including the helical domain of the Rab8 guanine nucleotide exchange factor Rabin8 (15) as well as phosphatidylinositol 4 kinase III β (PI4KB) (16). This noncanonical interface with Rab11 allows for formation of ternary complexes with FIP3 (Rabin8–Rab11–FIP3 or PI4KB–Rab11–FIP3). The Rab11–Rabin8–FIP3 complex increases the affinity of Rab11 for Rabin8, through both Rab11 and FIP3 directly binding Rabin8 (15). Formation of the Rab11–FIP3–Rabin8 complex plays critical roles in ciliogenesis through Rabin8 activation of Rab8 at ciliary membranes (6, 17). One of the first identified Rab11 effectors was the protein WDR44 (WD repeat–containing protein 44, also known as Rab11BP, or Rabphilin-11), but until recently, its exact role in Rab11 membrane trafficking has remained elusive (18, 19).

WDR44 is named for its C-terminal WD repeat domain, which forms a putative beta propeller domain. The Rab11-binding site was identified N-terminal to this region, with a region from residues 334 to 504 identified as the RBD (19). WDR44 localizes to tubular endosomes, which closely align to endoplasmic reticulum membranes, and acts as a scaffolding protein able to bridge the GRAF family of membrane tubulating proteins with Rab11 (20). Knockdown of WDR44 promotes ciliogenesis, with this putatively mediated by WDR44 competition for Rab11 binding with the prociliogenic FIP3–Rabin8 (21). The binding affinity of WDR44 for Rab11 can be increased upon phosphorylation of WDR44 by Sgk3 (22) or Akt (21) downstream of PI3K activation by serum lysophosphatidic acid. Lysophosphatidic acid treatment mediates blockade of preciliary trafficking of Rabin8–Rab11a, with phosphorylated WDR44 acting to block recruitment of FIP3–Rabin8. Hindering the ability to fully understand how WDR44 competes with FIP3 is the absence of any molecular information on its complex with Rab11.

In the present study, we determined the molecular mechanism of WDR44 binding to Rab11, and why it specifically binds to only Rab11 over other Rab GTPases. We modeled and validated the structure of this complex using a combined hydrogen/deuterium (H/D) exchange mass spectrometry (HDX-MS), AlphaFold2 modeling, and mutagenesis approach. We defined a minimal RBD of WDR44 and identified interfacial residues required for WDR44 binding. The WDR44 interface with Rab11 is distinct from the complex of FIP3 with Rab11, with HDX-MS used to contrast the dynamics of these complexes. Sgk3 efficiently phosphorylated WDR44 at S344 *in vitro* with phosphorylation stabilizing the RBD of WDR44. Together, our results reveal molecular mechanisms underlying Rab11–WDR44 binding, with important implications for understanding the role of Rab11 in ciliogenesis and membrane trafficking.

## Results

### Defining the minimum Rab11a-binding construct of WDR44

**Figure 1:**
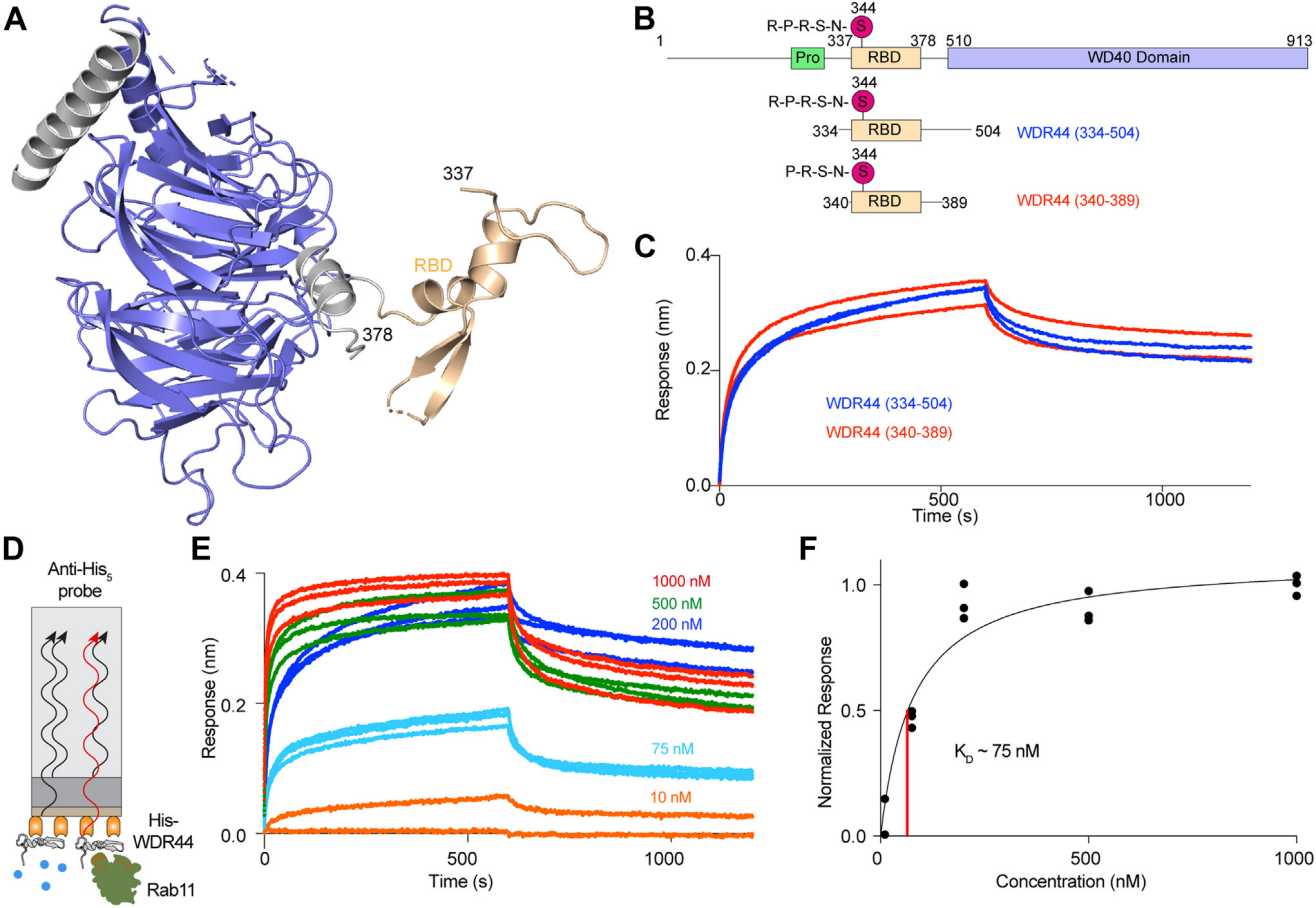
**Model of full-length WDR44 and defining the minimal RBD fragment and its affinity for Rab11.***A*, full-length WDR44 AlphaFold2 structure from the AlphaFold structure database (https://alphafold.ebi.ac.uk/) (23) with pLDDT <50 removed. Domains are colored according to *B* with the RBD colored in *tan*. Validation details are provided in Fig. S1*C*. *B*, the full-length domain schematic of WDR44 as well as fragments used to examine Rab11 binding. S344 is the site of phosphorylation. Domains are colored to match the AlphaFold structure in *A*. *C*, biolayer interferometry (BLI) association and dissociation traces of the shortest WDR44 (340–389) and longest WDR44 (334–504) fragments binding Rab11. Experiments were carried out using 25 nM or each WDR44 construct, with 150 nM of GTPγS-loaded Rab11. WDR44 protein was immobilized on the tip using a anti-His antibody (see schematic in *D*). *D*, *cartoon* schematic of BLI analysis of the binding of immobilized His-WDR44 (340–389) to Rab11a. *E*, association and dissociation curves for the dose response of His-WDR44 binding to varying Rab11 concentrations (10–1000 nM). Each experiment was carried out in triplicate, with all data graphed. *F*, normalized BLI response with varying concentrations of Rab11, with *K*_*D*_ estimated (*red line*) by a one-site binding nonlinear regression. Each data point is shown (n = 3). RBD, Rab-binding domain; WDR44, WD repeat–containing protein 44.

The WDR44 protein is structurally uncharacterized, with a Rab11a-binding domain spanning amino acids 334 to 408 initially identified through pull-down assays using a variety of WDR44 truncations (19). To provide additional insight into the assembly of WDR44, we examined the AlphaFold2 model of WDR44 (23), which showed a C-terminal WD40 repeat domain, with a small ordered region spanning the Rab11a-binding domain (Figs. 1*A* and S1). The RBD appeared to be dynamic relative to the WD40 domain, with no stable interfaces between the two domains. This suggested that using a fragment of the RBD should show similar binding to the full-length protein. Intriguingly, there was a predicted helix (383–392) located 10 amino acids from the C-terminal end of the RBD that packed against the WD40 domain (Fig. S1, *B*+*C*).

Using this initial information, we expressed and purified recombinant protein for WDR44 of varying lengths (Fig. S1*A*) and evaluated their binding using biolayer interferometry (BLI). SDS-PAGE gels showing purified protein for all constructs used in this article are highlighted in the source data. BLI experiments carried out using immobilized His-tagged WDR44 with an anti-His sensor tip showed that the shortest WDR44 truncation (340–389) bound with the same affinity compared with the longest WDR44 truncation (334–504) (Fig. 1*C*). This suggested that a minimal fragment of WDR44 that binds Rab11a was located between residues 340 and 389.

BLI was then used to determine the dissociation constant between Rab11a and WDR44 (Fig. 1*E*). The dissociation constant was determined to be ∼75 nM (Fig. 1*F*). This was consistent with previous binding kinetic experiments investigating the *K*_*D*_ of WDR44 (334–435) with Rab11a Q70L using isothermal titration calorimetry (21).

### HDX-MS and AlphaFold reveal molecular basis for WDR44–Rab11a complex formation

**Figure 2:**
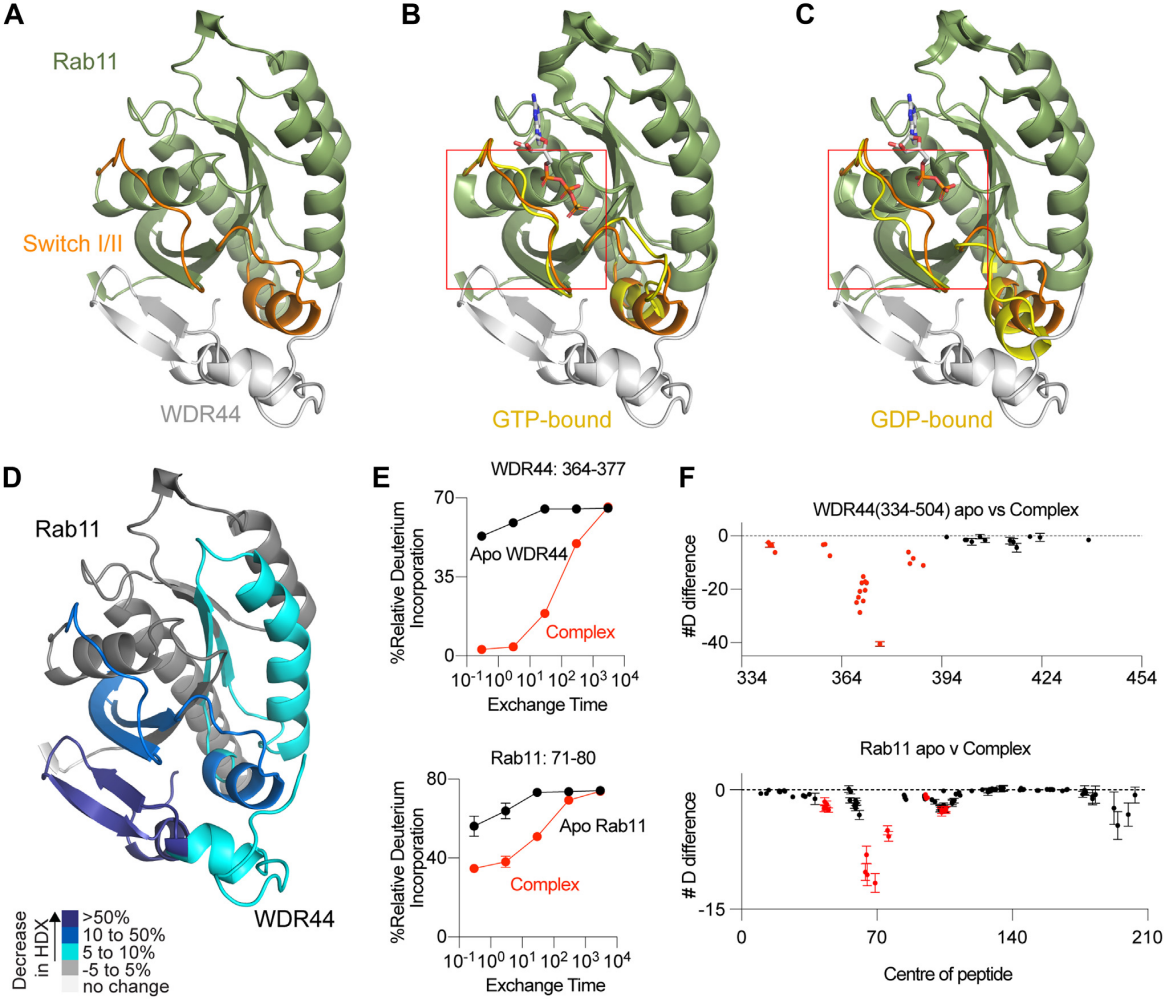
**Predicted Rab11–WDR44 complex and validation by HDX-MS.***A*, AlphaFold predicted complex structure of Rab11–WDR44 (334–392) with pLDDT <50 removed. Full methodological details of AlphaFold modeling are provided in Fig. S2. *B*, AlphaFold-predicted complex structure of Rab11–WDR44 (334–392) aligned to Rab11-GTPγS crystal structure (Protein Data Bank [PDB] ID: 1OIW) with structural differences highlighted in switch I. *C*, AlphaFold-predicted complex structure of Rab11–WDR44 (334–392) aligned to Rab11-GDP crystal structure (PDB ID: 1OIV) with structural differences highlighted in switch I. *D*, significant differences in HDX observed in either WDR44 (334–504) or Rab11 upon complex formation for all time points are mapped onto the AlphaFold structure. Differences in HDX are colored according to the legend. *E*, deuterium incorporation curves for selected peptides either apo (*black*) or in the WDR–Rab11a complex (*red*). Error is shown as standard deviation (n = 3). *F*, the number of deuteron differences for all analyzed peptides over the entire exchange time course for WDR44 and Rab11. Each point represents the central residue of an individual peptide. *Red* indicates peptides with a significant difference (defined as >5% change in exchange and a >0.5 Da difference in exchange at any time point, along with a *p* < 0.01 using a two-tailed Student's *t* test), *black* indicates no significant difference between apo and complex. For all panels, error bars show SD (n = 3). HDX-MS, hydrogen/deuterium exchange mass spectrometry; WDR44, WD repeat–containing protein 44.

Using the ColabFold AlphaFold2 advanced notebook (24, 25), we generated three different WDR44–Rab11a predicted models using WDR44 constructs of different lengths (340–389, 334–389, and 334–392) with full-length Rab11. All searches resulted in heterodimer complexes that had excellent statistics for the per-residue confidence and pLDDT scores indicative of a high-confidence solution for the protein–protein interface (Fig. S2). The prediction with the highest confidence was the WDR44 (334–392)–Rab11a complex and is utilized in all subsequent figures. As the AlpahFold2 searches were carried out in the absence of nucleotide, we compared the resultant search to structures of Rab11 bound to different nucleotides. The predicted WDR44–Rab11a structure overlaps more closely with the crystal structure of Rab11 bound to GTPγS (Protein Data Bank [PDB] ID: 1OIW) than the crystal structure of Rab11 bound to GDP (PDB ID: 1OIV), which is consistent with WDR44 preferentially binding GTP-Rab (26). There are structural differences in the switch I region between GTPγS and GDP-bound Rab11a, with the GDP-bound version putatively clashing with WDR44 (Fig. 2*A*). The predicted structure of the WDR44 RBD (334–389) consists of a loop (334–350) and two short alpha helices (α1–α2) that sandwich a central beta sheet (358–369). The putative Rab11a-binding interface of WDR44 is composed of residues in helix α1, the central beta sheet, and helix α2, with the WDR44-binding interface of Rab11a composed of residues at the N terminus, residues in switch I and II, the interswitch region, and part of the C-terminal helix. The interface is composed of a large primarily hydrophobic surface (∼1050 Å^2^) with extensive contacts between both switch I and II. The putative hydrophobic contact residues of Rab11a include well-conserved residues important for Rab–effector complex formation (27).

To further investigate the WDR44–Rab11a structure, we carried out HDX-MS experiments of WDR44 and Rab11a to validate our predicted model and investigate the interface between WDR44 and Rab11a. HDX-MS measures the deuterium incorporation of amide hydrogens, with the exchange rate being dependent on secondary structure stability (28, 29). It is a powerful tool to investigate conformational changes and define protein–protein interfaces. We utilized WDR44 (334–504) with full-length Rab11a loaded with GTPγS. Deuterium incorporation experiments required generation of pepsin peptide fragments, with peptide maps covering 64% of WDR44 and 98% of Rab11a (Tables S1–S3). There was 100% sequence coverage of the Rab11a-binding region (334–389) of WDR44, with poor coverage of the disordered C terminus (∼437–504). Significant differences in exchange between conditions were defined as differences at any time point fitting the following three criteria (a difference >5% and >0.5 Da, with a two-tailed *t* test; *p* < 0.01).

Initial HDX experiments were carried out comparing three conditions: apo-WDR44, apo-Rab11a, and WDR44–Rab11a complex. We observed decreases in exchange throughout WDR44 (334–399) upon Rab11a binding and in Rab11a (37–120) upon WDR44 binding (Fig. 2*C*). Decreases in WDR44 mapped primarily to the Rab11a-binding site with no significant changes in the disordered C terminus of the construct (400–504). Regions with largest decreases (>50%) in WDR44 spanned peptides from 358 to 391, which correspond to the end of the first alpha helix and into the beta sheet of WDR44 (Fig. 2*D*), which formed extensive contacts with Rab11a in the AlphaFold2 model. Decreases in Rab11a spanned switch I, interswitch, and switch II regions (37–90), which is consistent with effectors binding to Rab GTPases (2, 3). The Rab11a regions with the largest decreases (>20%) upon WDR44 binding spanned peptides covering switch I and II (Fig. 2*D*). All changes were consistent with the predicted AlphaFold2 model and reveals initial insight into the orientation of the Rab11a–WDR44 complex.

### Mutational analysis of WDR44 and Rab11a highlights key residues at the interface

**Figure 3:**
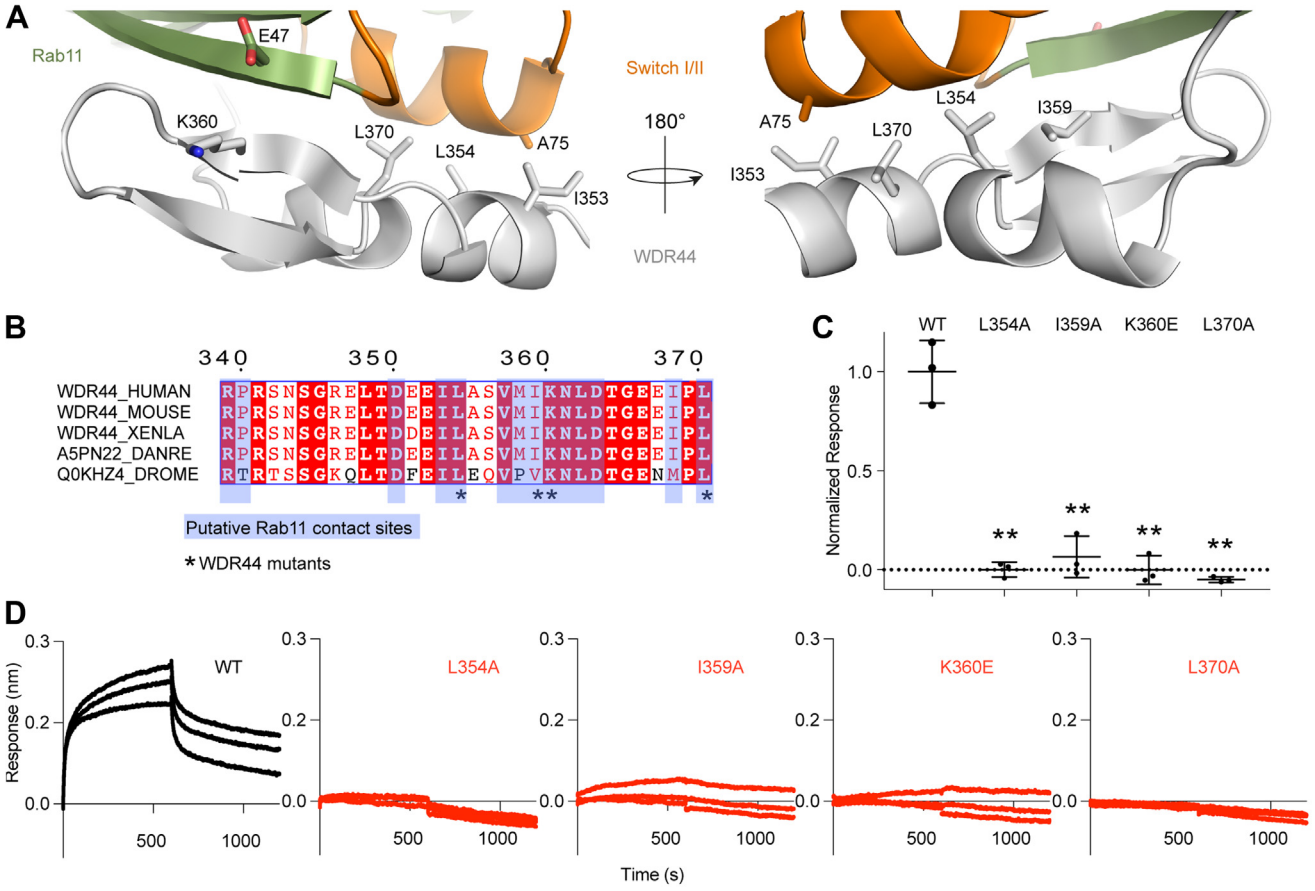
**Validation of the WDR44–Rab11a interface.***A*, zoomed in view of the Rab11–WDR44 model with interfacial residues labeled. Rab11 is colored in *smudge* with switch I/II colored in *orange* and WDR44 in *silver*. *B*, alignment of WDR44 Rab-binding domain between eukaryotic model organisms (*Homo sapiens*, *Mus musculus*, *Xenopus laevis*, *Danio rerio*, and *Drosophila melanogaster*). Putative contact residues within 4 Å of Rab11 determined using the AlphaFold complex structure are colored *blue*. Evolutionarily conserved residues that were mutated and tested in *C* and *D* are highlighted with an ∗. *C*, normalized BLI responses of various WDR44 mutants binding Rab11 (25 nM WDR44, with 75 nM Rab11). Responses were normalized to the mean wildtype response. Two-tailed *p* values are represented as follows: ∗∗<0.001 and ∗<0.01. Error bars represent SD (n = 3). *D*, raw BLI association and dissociation curves of each mutant binding Rab11. About 25 nM WDR44 (334–389) mutants were immobilized onto fiber optic anti–penta-His tips and dipped into Rab11 at 75 nM. BLI, biolayer interferometry; WDR44, WD repeat–containing protein 44.

To further validate the prediction of the WDR44–Rab11a complex and of the WDR44 Rab-binding site, we carried out extensive mutagenesis experiments. The WDR44 region with the largest differences in H/D exchange upon Rab11a binding spanned amino acids 358 to 391 (Fig. 2*D*). Utilizing this along with the AlphaFold2 model, as well as comparing evolutionarily conserved residues in WDR44, we found several putative amino acids lying at the interface with Rab11a. These include hydrophobic residues in WDR44 in helix α1 (L354), which contacts switch II, the central beta sheet (I359), which contacts switch I, and helix α2 (L370), which contacts switch II, and a putative salt bridge between K360 of WDR44 and E47 of switch I. We mutated the hydrophobic residues (L354A, I359A, and L370A) and charged residues (K360E) at the Rab11a interface (Fig. 3*A*), with these resulting in a >95% decrease in WDR44–Rab11a complex formation detected by BLI (Fig. 3, *C*+*D*). Together with the HDX-MS, this provides additional validation of the AlphaFold2 model.

### Differences between WDR44 and FIP3 binding to Rab11a

**Figure 4:**
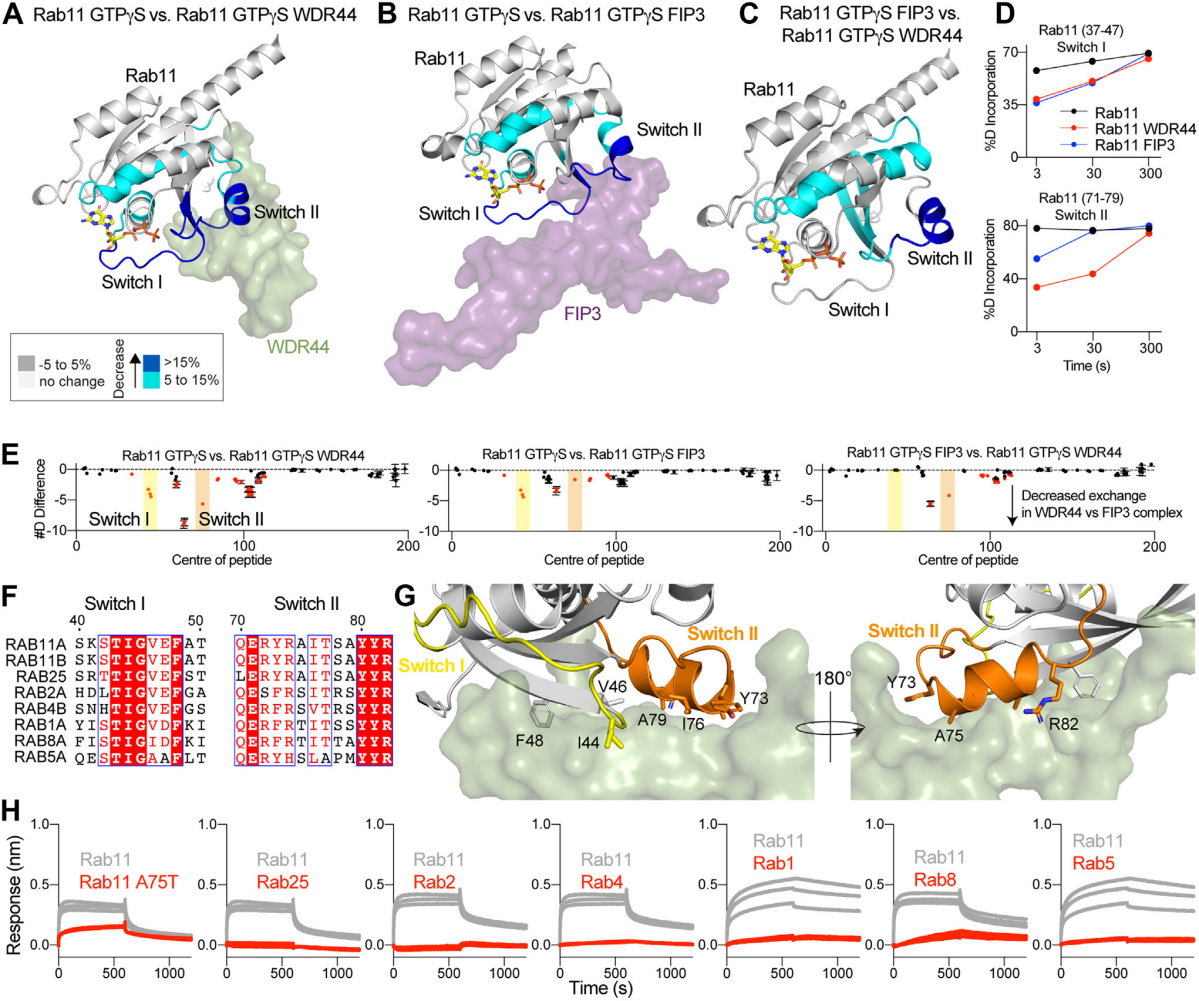
**HDX shows differences between WDR44 and FIP3 binding Rab11.***A*, significant differences in HDX observed in Rab11 upon binding WDR44 (334–389) are mapped onto the AlphaFold structure. Differences in HDX are colored according to the legend. *B*, significant differences in HDX observed in Rab11 upon binding FIP3 (713–756) are mapped onto the crystal structure (PDB ID: 2HV8). Differences in HDX are colored according to the legend. *C*, significant differences in HDX between WDR44 (334–389) and FIP3 (713–756) binding Rab11 are mapped onto the crystal structure (PDB ID: 1OIW). Differences in HDX are colored according to the legend. *D*, deuterium incorporation curves for peptides spanning switch I (37–47) and switch II (71–79) upon complex formation with WDR44 and FIP3 compared with apo-Rab11. Error bars report standard deviation (n = 3) with most being smaller than the size of the point. *E*, the number of deuteron differences for all analyzed peptides over the entire exchange time course for Rab11 in the different states indicated. Each point represents the central residue of an individual peptide. *Red* indicates a peptide with a significant difference (defined as >5% change in exchange and a >0.5 Da difference in exchange at any time point, along with a *p* < 0.01 using a two-tailed Student's *t* test), *black* indicates no significant difference. Error bars show SD (n = 3). *F*, alignment of switch I and II residues for a subset of human Rab GTPases. *G*, zoomed in view of the Rab11–WDR44 (334–392) AlphaFold model with interfacial Rab11 residues highlighted and WDR44 shown as a *green* colored surface. *H*, biolayer interferometry association and dissociation curves of each replicate. About 25 nM WDR44 (334–389) were immobilized onto fiber optic tips and dipped into Rab GTPases at 500 nM. Rab11 is shown in *gray* for each Rab tested. FIP3, family of Rab11-interacting protein 3; HDX, hydrogen/deuterium exchange; PDB, Protein Data Bank; WDR44, WD repeat–containing protein 44.

The predicted structure of Rab11a bound to WDR44 revealed a distinct interface compared with the previously determined Rab11a-FIP3 complex (10, 11). To further investigate the dynamic differences between the WDR44–Rab11a complex and other effector complexes, we carried out HDX-MS experiments comparing WDR44–Rab11a to FIP3–Rab11a. We analyzed the following conditions: apo-Rab11a, WDR44–Rab11a, and FIP3–Rab11a, which allowed us to map changes upon complex formation in Rab11a and compare any significant differences in Rab11a between the two complexes. There was decreased deuterium incorporation in Rab11a upon WDR44 binding in regions spanning switch I and II and the helix that switch II packs up against (89–199) (Fig. 4, *A*+*D*). Decreases in exchange upon FIP3 binding spanned similar regions of Rab11a, although with altered dynamics (Fig. 4, *B*+*D*). Interestingly, when we compare the significant differences between the WDR44–Rab11a and FIP3–Rab11a complexes, Rab11a was more protected in switch II (71–79) and the helix that switch II packs against (89–100) in the WDR44 complex compared with FIP3. Intriguingly, switch I of Rab11a showed almost the same decreases for both WDR44 and FIP3 (Fig. 4*D*). Comparing Rab11a–FIP3 with Rab11a–WDR44 revealed a more extensive interface in the switch II region for WDR44, which is consistent with the HDX-MS data. Overall, this suggests that both FIP3 and WDR44 make similar contacts with switch I, with WDR44 making more extensive interactions at switch II compared with FIP3.

### Exploring the molecular basis for specificity of the Rab11a–WDR44 interaction

To provide further insight into the specificity of the Rab11a–WDR44 interface, we carried out BLI assays for WDR44 binding to a set of conserved Rab GTPases (Rab1a, Rab2a, Rab4b, Rab5a, Rab8a, and Rab25). None of these Rabs showed any significant binding to WDR44 (Fig. 4*H*), even the very evolutionarily similar Rab25 isoform. While a major factor in Rab11a effector binding is the differential plasticity of the switch regions compared with other Rab GTPases (3, 27), we also wanted to investigate if there were any specific residues in Rab11a that play a role in the specificity of this interaction. We analyzed the evolutionary conservation of switch II for a variety of other Rab GTPases and noted a conserved alanine residue (A75) in Rab11a/b and Rab25 that sits in a hydrophobic pocket of the WDR44 interface (Fig. 4, *F*+*G*). We hypothesized that mutating this to a larger amino acid (serine or threonine as in other nonbinding Rab GTPases tested) may inhibit binding to WDR44. Mutant A75T Rab11 significantly disrupted binding to WDR44, likely through steric clashes between switch II and WDR44 (Fig. 4*H*).

### HDX-MS shows phosphorylation stabilizes WDR44 at the Rab11a-binding interface

**Figure 5:**
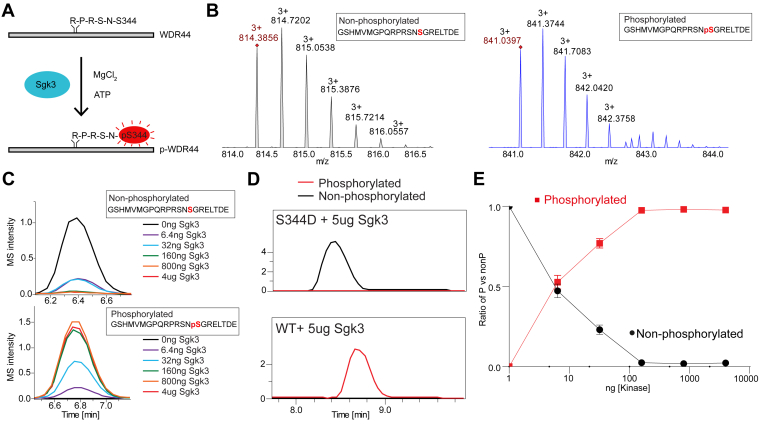
**WDR44 can be phosphorylated efficiently by Sgk3 specifically at S344.***A*, schematic of WDR44 (334–504) phosphorylation of S344 *in vitro* using Sgk3. *B*, peptide spectra of the unphosphorylated (*left*) and phosphorylated (*right*) WDR44 peptide, with S344 highlighted in *red* in the sequence. *C*, graph showing the intensity of unphosphorylated (*top*) and phosphorylated (*bottom*) peptide according to a dose response of Sgk3. *D*, MS intensity of phosphorylated (*red*) and unphosphorylated (*black*) peptide for wildtype and S344D WDR44 treated with 5 μg Sgk3. *E*, ratio of phosphorylated to unphosphorylated WDR44 graphed against Sgk3 kinase concentration from data in *C*. MS, mass spectrometry; WDR44, WD repeat–containing protein 44.

It has been proposed that WDR44 can be phosphorylated at S342–S344 by Akt with this disrupting ciliogenesis by preventing the formation of the prociliogenic Rab11a–FIP3–Rabin8 complex (21). However, it was also proposed that WDR44 is phosphorylated preferentially by Sgk3 at only S344 as Akt prefers a bulky hydrophobic residue at the n + 1 position, with WDR44 containing a glycine residue at the n + 1 position (22). To define conformational changes accompanying phosphorylation of WDR44, we treated WDR44 with Sgk3 (Fig. 5, *A*+*C*). WDR44 was efficiently phosphorylated by Sgk3 as tracked by MS (Fig. 5*C*), with a figure showing the MS/MS spectra of phosphorylated peptides shown in Fig.S3. Phosphorylation was only detected in peptides containing S344, although because of the inherent issue of collision-induced dissociation fragmentation leading to loss of phosphate in b-y fragments, there was ambiguity in the phosphorylation site in the MS data. To further validate the Sgk3 phosphorylation site being S344, we tested the phosphorylation of a S344D mutant, which showed no detectable phosphorylation upon Sgk3 treatment (Fig. 5*D*).

**Figure 6:**
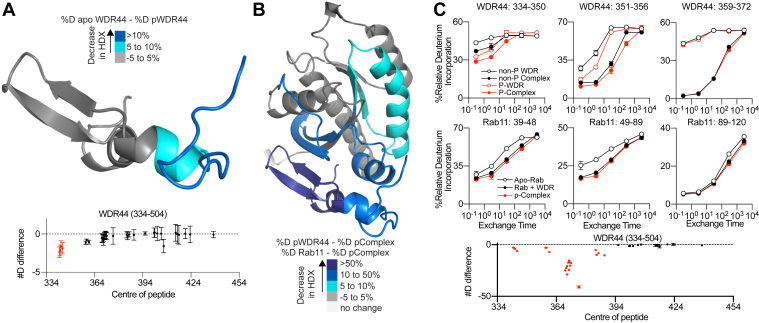
**Phosphorylation of WDR44 stabilizes part of the Rab11-binding interface of WDR44.***A*, significant differences in HDX upon phosphorylation of apo-WDR44 are mapped onto the AlphaFold model of WDR44 alone according to the legend (*top*). The number of deuteron differences for all analyzed peptides over the entire exchange time course for WDR44 compared with phosphorylated WDR44. Each point represents the central residue of an individual peptide. *Red* indicates a peptide with a significant difference, and *black* indicates no significant difference. For all panels, error bars show SD (n = 3) (*bottom*). *B*, significant differences in HDX upon Rab11 incubation with phosphorylated WDR44 (334–504) are mapped onto the AlphaFold model according to the legend. *C*, deuterium incorporation curves for selected WDR44 and Rab11 peptides highlighting the differences upon complex formation with phosphorylated and unphosphorylated WDR44 (*top*). The number of deuteron differences for all analyzed peptides over the entire exchange time course for phosphorylated WDR44 compared with phosphorylated WDR44 in complex with Rab11. Each point represents the central residue of an individual peptide. *Red* indicates a peptide with a significant difference, and *black* indicates no significant difference. Error bars show SD (n = 3) (*bottom*). HDX, hydrogen/deuterium exchange; WDR44, WD repeat–containing protein 44.

We carried out HDX-MS experiments on stoichiometrically phosphorylated WDR44 (>99% as measured by MS) compared with unphosphorylated WDR44 in the presence and absence of Rab11a. HDX-MS experiments compared five different conditions: apo-WDR44, phosphorylated WDR44, apo-Rab11a, WDR44–Rab11a complex, and phosphorylated WDR44–Rab11a complex. Comparing apo-WDR44 *versus* phosphorylated WDR44 showed decreased exchange for phosphorylated WDR44 in peptides spanning the N terminus of the RBD (334–354) (Fig. 6, *A*+*C*). It is worth noting that some of these differences in deuterium incorporation between apo and phosphorylated WDR44 may be caused by phosphorylation altering the intrinsic exchange rate of amide hydrogens; however, we also see a protection between apo and phosphorylated on a peptide that does not contain the phosphorylation site (WDR44: 351–356) (Fig. 6*C*), suggesting that this decreased exchange is driven by conformational changes driven by phosphorylation. Similar decreases in exchange were observed for phosphorylated WDR44 binding Rab11a compared with unphosphorylated WDR44 binding Rab11a (Fig. 6, *B*+*C*). Intriguingly, the region protected by phosphorylation in WDR44 is in the Rab11a interface, revealing that phosphorylation restructures the Rab11a-binding interface in WDR44.

## Discussion

The formation of Rab11a–effector complexes plays critical roles during membrane trafficking processes, including regulating the recycling endosomal network (5) and in ciliogenesis (21). The Rab11a–WDR44 complex inhibits ciliogenesis through disruption of the FIP3–Rabin8 complex. However, the molecular details regarding how Rab11a interacts with WDR44 is poorly understood, particularly any differences compared with other Rab11 effectors. Our structural and biochemical investigation of the Rab11a–WDR44 complex has identified interfacial residues and dynamic changes that occur upon both Rab11 binding and Sgk3 phosphorylation of WDR44. Overall, this provides fundamental insight into how Rab11 recognizes its downstream effectors as well as how phosphorylation can alter this recognition.

WDR44 was one of the first identified Rab11 effectors (18, 19), but its roles in regulating Rab11-dependent processes have only recently begun to be appreciated. The AlphaFold2 model (24) of the full-length WDR44 complex showed that the RBD of WDR44 appears to be dynamic relative to the C-terminal WD40 solenoid domain. However, there is only a short dynamic 10 amino acid stretch between a helix that packs against the WD40 domain, and the beginning of the RBD, which opens the possibility of some regulation of Rab11 binding in the full-length protein, with further structural studies required to explore this interface. Using a combined HDX-MS and AlphaFold2 modeling approach allowed us to make a model of the Rab11a–WDR44 complex. Similar to many other Rab11 effectors, the primary interface was composed of switch I + II and the interswitch region. There is a large degree of structural plasticity of the switch regions of Rab11 with its different effector proteins, particularly in switch II, with this region having unique conformations in Rab11a-GTP bound to FIP3, myosin V, and PI4KB (10, 13, 16). This is unique from other Rab GTPases, which do not show the same switch plasticity upon binding effectors (7, 30). The interface of WDR44 with Rab11a from the AlphaFold model showed a unique orientation of switch II compared with other Rab11 effectors, although because of the absence of nucleotide in the AlphaFold2 modeling, further structural studies will be needed to validate this specific conformational change. The Rab11a–WDR44 model did show a much more extensive interface with switch II compared with the Rab11a–FIP3 structure (10), which is corroborated by our HDX-MS data showing enhanced protection in switch II for WDR44 compared with FIP3.

The Rab11 interface in WDR44 is highly conserved through evolution, with mutation of both hydrophobic and electrostatic interactions leading to almost complete abrogation of the Rab11–WDR44 complex. We mutated residues in all three predicted secondary structure elements of WDR44 in contact with Rab11, with all three being critical in complex assembly. The generation of complex disruption mutants is essential in fully defining the role of WDR44 in ciliogenesis. This is due to the fact that the PI3K–Akt pathway can alter ciliogenesis by multiple routes. Phosphorylation of WDR44 by Akt or Sgk3 downstream of Akt can alter the binding affinity for Rab11 (21, 22), preventing recruitment of FIP3 and Rabin8 (15) and decreasing Rab8 trafficking. Akt phosphorylation of Rabin8 can also putatively inhibit guanine nucleotide exchange factor activity, also leading to decreased Rab8 trafficking (21). There is also the potential of unexpected crosstalk between PI3K signaling and Rabin8, as the class IB PI3K isoform in immune cells can be activated by GTP-Rab8 downstream of Toll-like receptor signaling, potentially complicating analysis of the role of PI3K–Akt activation in regulation of either WDR44 *versus* Rabin8 (31). These mutants will act as important tools that can be used in further studies defining the role of the specific WDR44–Rab11 complex in ciliogenesis.

We found that WDR44 was efficiently phosphorylated by Sgk3 using an *in vitro* approach, consistent with previous results (22). This allowed us to generate stoichiometrically phosphorylated recombinant WDR44 for analysis by HDX-MS. Phosphorylation stabilized the N terminus of the RBD, including regions that interact directly with Rab11. It is well appreciated that phosphorylation of Rab GTPases can dramatically alter their roles in membrane trafficking (32), with the Parkinson-associated kinase LRRK2 regulating a subset of GTPases through direct phosphorylation (33, 34). One of the putative roles of LRRK2 is through regulating ciliogenesis by enhancing the interaction of phosphorylated Rab8 with its effectors RILPL1/2 (34, 35). Further studies of phosphorylated full-length WDR44 will allow for structural and biochemical studies to define the exact role of WDR44 phosphorylation in regulating Rab11 recruitment and binding.

Rab GTPases play crucial roles in nearly all steps of membrane trafficking by recruiting specific effectors depending on their nucleotide-bound state. The formation of the Rab11–WDR44 complex plays an important role in ciliogenesis, but its study has been hindered by lack of details on its molecular assembly. Our data highlight the molecular basis for complex formation, how it has been conserved over evolution, and its differential dynamics from other Rab11–effector complexes. Critically, WDR44 mutants developed in this study can be used in future *in vivo* experiments to study the function of WDR44 and Rab11 in membrane trafficking and ciliogenesis.

## Experimental procedures

**Table:** 

Key resources	Source	Identifier
Bacterial or viral strains		
*Escherichia coli* XL10-GOLD KanR ultracompetent cells	Agilent	200317
OverExpress C41(DE3) chemically competent cells	MilliporeSigma	CMC0017
Chemicals, peptides, and recombinant proteins		
Deuterium oxide 99.9%	Sigma–Aldrich	151882-10X1ML
GTPγS tetralithium salt	Sigma–Aldrich	G8634
ATP	Sigma	A7699-1g
MgCl_2_ ∗ 6H_2_O	Bio Basic	7791-18-6
Recombinant DNA		
WDR44 (*Homo sapiens*) in pDONR221	DNASU	hsCD00296048
Strep-His WDR44 (334–504)	This article	NH17
WDR44 (334–504) cleaved	This article	NH22
WDR44 (334–504) glutathione-*S*-transferase	This article	NH22
Strep-His WDR44 (334–389)	This article	NH63
Strep-His WDR44 (340–389)	This article	MJ234
Strep-His WDR44 (334–389) L354A	This article	MT12
Strep-His WDR44 (334–389) I359A	This article	MT15
Strep-His WDR44 (334–389) K360E	This article	MT14
Strep-His WDR44 (334–389) L370A	This article	MT11
WDR44 (334–389) S344D	This article	NH64
Rab11a	PMID: 33277614	MF19
Rab11a Q70L		JB88
Rab11a Q70L A75T	This article	MT16
Rab25	This article	MT17
Rab2a	PMID: 33277614	MJ31
Rab4b	PMID: 33277614	MJ30
Rab1	PMID: 33277614	MJ118
Rab5a	PMID: 33277614	DH1
Sgk3	PMID: 34181950	Sgk3
Software and algorithms		
HDExaminer	Sierra Analytics	http://massspec.com/hdexaminer/
GraphPad Prism 7	GraphPad	https://www.graphpad.com/scientific-software/prism/
PyMOL	Schrödinger	https://pymol.org/
Adobe Illustrator 2022	Adobe	https://www.adobe.com/products/illustrator.html
Other		
Octet HIS1K Biosensors	Sartorius	18-5120

### Plasmids and antibodies

The full-length Rab genes were used as previously described (36, 37). The WDR44 gene was purchased from the DNASU plasmid repository (HsCD00296048). All WDR44 constructs were designed and subcloned into a tobacco etch virus (TEV)–cleavable N-terminal 2xStrep 10xHis-tagged vector, with all Rab constructs containing an N-terminal TEV-cleavable glutathione-*S*-transferase (GST) affinity tag. A figure summarizing plasmid used in this article is outlined in Fig.S1.

### Bioinformatics

Sequences were aligned using Clustal Omega Multiple Sequence Alignment, and the aligned sequences were subsequently analyzed by ESPript 3.0 (https://espript.ibcp.fr) (38) to visualize conserved regions. The UniProt accession codes for the aligned sequences used in Figures 3 and 4 are Q5JSH3, Q6NVE8, Q498F0, A5PN22, Q0KHZ4, P62491, Q15907, P57735, P61019, P61018, P62820, P61006, and P20339.

### AlphaFold

AlphaFold is an artificial intelligence software developed by DeepMind that predicts protein structures from a primary amino acid sequence (24). AlphaFold Advanced 2.0 Google Colab (https://colab.research.google.com/github/sokrypton/ColabFold/blob/main/beta/AlphaFold2_advanced_beta.ipynb) (25) was used to model the structure of Rab11–WDR44 without templates. The sequences of full-length Rab11a Q70L alongside three different constructs of WDR44 were input to generate the three best models. The WDR44 constructs used were residues 340 to 389, 334 to 389, and 334 to 392. For each input, AlphaFold generated five models ranked in order by mean pLDDT. The predicted aligned error for each output and the overlain structures are highlighted in Fig. S2. AlphaFold-Multimer was used to validate the structural model from which the results were very similar (data not shown) (39). The best structural model that was used for the HDX-MS color mapping and main text figures is included with the article as an attached PDB file, and residues with Cα pLDDT <50 were removed.

### Protein expression

Rab11 and WDR44 constructs were expressed in BL21 DE3 C41 *Escherichia coli* and induced with 0.5 mM (4 h) or 0.1 mM (18 h) IPTG and grown at 37 °C for 4 h or 21 °C for 18 h. Cells were then centrifuged at 15,000*g*. Pellets were washed with ice-cold PBS, flash frozen in liquid nitrogen, and stored at −80 °C for later use.

### Protein purification

For WDR44 purification, cell pellets were lysed by sonication for 5 min in lysis buffer (20 mM Tris [pH 8.0], 100 mM NaCl, 5% [v/v] glycerol, 20 mM imidazole, 2 mM β-mercaptoethanol [bME], and protease inhibitors [Millipore Protease Inhibitor Cocktail Set III, animal-free]). Triton X-100 was added to 0.1% v/v, and the solution was centrifuged for 45 min at 20,000*g* at 1 °C. The supernatant was then loaded onto a 5 ml HisTrap column (GE Healthcare) that had been equilibrated in nickel–nitrilotriacetic acid (Ni–NTA) A buffer (20 mM Tris [pH 8.0], 100 mM NaCl, 20 mM imidazole [pH 8.0], 5% [v/v] glycerol, and 2 mM bME). The column was washed with 15 ml of high salt buffer (20 mM Tris [pH 8.0], 1 M NaCl, 5% [v/v] glycerol, and 2 mM bME), followed by 15 ml of 6% Ni–NTA B buffer (20 mM Tris [pH 8.0], 100 mM NaCl, 200 mM imidazole [pH 8.0], 5% [v/v] glycerol, and 2 mM bME) before being eluted with 15 ml of 100% Ni–NTA B. The eluate was then loaded twice onto a 5 ml Strep column and then washed with 10 ml gel filtration buffer (GFB) (20 mM Hepes [pH 7.5], 150 mM NaCl, and 1 mM Tris(2-carboxyethyl)phosphine (TCEP). Protein was eluted with 10 ml GFB containing 2.5 mM desthiobiotin. Protein fractions were pooled and concentrated using an Amicon 3 K concentrator. Protein was loaded onto a 5 ml desalting column to remove desthiobiotin and eluted with 2 ml GFB. Eluate was further concentrated in a 3 K concentrator. Purified protein was flash frozen in liquid nitrogen and stored at −80 °C.

For Rab11 purification, cell pellets were lysed by sonication for 5 min in lysis buffer (20 mM Tris [pH 8.0], 100 mM NaCl, 5% [v/v] glycerol, 20 mM imidazole, 2 mM bME, and protease inhibitors [Millipore Protease Inhibitor Cocktail Set III, animal-free]). Triton X-100 was added to 0.1% v/v, and the solution was centrifuged for 45 min at 20,000*g* at 1 °C. Filtered supernatant was superloop loaded for 45 min onto a 5 ml GSTrap column, which was equilibrated with HepA buffer (20 mM Tris [pH 8.0], 100 mM NaCl, 5% [v/v] glycerol, and 2 mM bME). The GST tag was cleaved by adding a HepA buffer containing 2 mM bME and TEV protease to the column and incubating overnight at 4 °C. Cleaved protein was eluted with HepA buffer. Protein was further purified by separating on a 5 ml HiTrap Q column and eluting with 10 ml HepA buffer containing 2 mM bME. Protein was pooled and concentrated using an Amicon 10 K concentrator and flash frozen in liquid nitrogen and stored at 80 °C.

Rab11 was loaded with GTPγS before being used in various BLI assays. About 5 mM EDTA was incubated with protein in GFB (20 mM Hepes [pH 7.5], 150 mM NaCl, and 1 mM TCEP) for 30 min at 25 °C before being centrifuged at 15,000*g* for 1 min. About 10-fold excess GTPγS was added to the protein and incubated for 60 min at 25 °C before being centrifuged at 15,000*g* for 1 min. About 10 mM MgCl_2_ was added and incubated for 30 min at 25 °C. The protein mixture was then centrifuged at 15,000*g* for 5 min. Size-exclusion chromatography was performed using a Superdex 75 Increase 10/300 GL column, which was equilibrated in GFB with 1 mM MgCl_2_. Fractions were collected and concentrated using an Amicon 10K concentrator and flash frozen in liquid nitrogen and stored at −80 °C.

Sgk3 used to phosphorylate WDR44 was purified as previously described (40). The human Sgk3 gene was codon optimized for expression in insect cells (Genscript) and fused to the C terminus of His10/StrepII-tagged enhanced GFP (A206 K monomeric variant) to aid expression of soluble Sgk3. Sgk3 was coexpressed with phosphoinositide-dependent kinase 1 (PDK1) using a pFastBac Dual vector in baculovirus-infected Sf9 insect cells grown in ESF 921 medium (Expression Systems) at 27 °C. Bacmids were prepared from *E. coli* DH10EMBacY cells containing a modified bacmid for titer determination of baculovirus using YFP expressed from a polyhedrin promoter in the baculoviral genome. Sf9 cells were transfected with bacmid using Fugene (Promega), and two rounds of viral amplification were performed in 2 ml adherent (1 × 10^6^ cells/well) and 25 ml suspension (1 × 10^6^ cells/ml) cultures to generate a V1 virus. For infection of Sf9 cells for recombinant protein expression, suspension cells (1 l) were grown to a density of 2 × 10^6^ cells/ml in Schott Duran 2 l flasks before infection with 2 ml of V1 virus. Cells grew at 27 °C, shaking at 100 rpm until viability was approximately 80%. Cells were typically harvested 4 to 5 days postinfection. The pellet of Sf9 cells was resuspended in 50 ml Sgk3 lysis buffer: 50 mM Hepes (pH 7.4), 50 mM NaCl, 1% (v/v) glycerol, 1 mM TCEP, 1 mM PMSF, 1 mM benzamidine hydrochloride, 1 mM MnCl_2_, 0.5 mM EDTA, 1× protease inhibitor cocktail, 0.1% CHAPS, 5 μl DENARASE, 10 mM DTT, 1× protease inhibitor cocktail, 5 μl DENARASE, 80 nM lambda phosphatase (made in-house). The lysate was passed through a Dounce homogenizer several times and spun at 38,724*g* (18,000 RPM), 4 °C for 30 min. The soluble fraction was loaded onto a StrepTrap HP 5 ml column (Cytiva) equilibrated in 50 mM Hepes (pH 7.4), 50 mM NaCl, 1% (v/v) glycerol, and 1 mM TCEP. The column was washed with 10 column volumes of binding buffer, and the protein was eluted with 2.5 mM d-desthiobiotin in binding buffer. Fractions containing enhanced GFP-Sgk3 were pooled and dephosphorylated with lambda phosphatase in 50 mM Hepes (pH 7.4), 150 mM NaCl, 1% (v/v) glycerol, 10 mM DTT, 1 mM MnCl_2_, 0.5 mM EDTA, and 0.5 μM lambda phosphatase (made in-house). Dephosphorylation was carried out for 30 min at room temperature and 4 °C overnight together with TEV cleavage of the His10/StrepII tag. To separate His-tagged lambda phosphatase from Sgk3, the reaction mixture was loaded on HisTrap FF 1 ml column equilibrated in 20 mM Tris (pH 7.4), 150 mM NaCl, 1% (v/v) glycerol, and 1 mM TCEP. Dephosphorylated and cleaved Sgk3 was washed from the column with 20 mM imidazole, whereas lambda phosphatase remained bound. Sgk3 was then site-specifically *in vitro* rephosphorylated by GST-PDK1 under the following conditions: 50 mM Tris (pH 7.4), 150 mM NaCl, 1% (v/v) glycerol, 1 mM TCEP, 1 mM ATP, 2 mM MgSO_4_, and 1:10 PDK1:Sgk3. The reaction was carried out at 4 °C for 3 h and then stopped by addition of 10 mM EDTA. GST-PDK1 was separated by addition of glutathione-sepharose 4B beads (Cytiva) and incubation at 4 °C overnight with gentle rotation. Supernatant containing rephosphorylated Sgk3 was concentrated in 30 kDa molecular weight cutoff spin concentrator and injected onto a Superdex 200 Increase 10/300 GL column (Cytiva) equilibrated in 20 mM Tris (pH 7.4), 150 mM NaCl, 1% (v/v) glycerol, and 1 mM TCEP. Fractions containing Sgk3 were pooled, concentrated, snap-frozen in LN2, and stored at –80 °C for further use.

### Phosphorylation of WDR44 by Sgk3

For the dose–response phosphorylation of WDR44 (334–504), WDR44 was mixed with ATP, GFB (20 mM Hepes [pH 7.5], 150 mM NaCl, 0.5 mM TCEP, and 5% glycerol), and MgCl_2_. Sgk3 was serially added to WDR44 (334–504) at 4 μg, 800 ng, 160 ng, 32 ng, 6.4 ng, and 0 ng to start the reaction. Final concentrations were WDR44 (2 μM), Sgk3 at 2.4 μM, 0.48 μM, 0.095 μM, 0.019 μM, 0.0038 μM, 0.00076 μM, ATP (200 μM), and MgCl_2_ (20 mM), diluted in buffer. Reactions were incubated for 45 min at room temperature (21 °C) followed by 17 h incubation on ice and quenching with 10 mM EDTA followed by immediate freezing using liquid nitrogen and storage at −80 °C. Phosphorylation was confirmed using MS (full MS quantification of each of the phosphorylated and nonphosphorylated peptide is provided in the source data). For mutant S344D phosphorylation, WT WDR44 (108 μM) and WDR44 S344D (167 μM) were mixed with ATP (800 μM) and MgCl_2_ (20 mM), and reactions were started by adding Sgk3 (2.9 μM). Reactions were incubated at room temperature for 45 min followed by 17 h incubation on ice, and quenching with 10 mM EDTA followed by immediate freezing using liquid nitrogen, and storage at −80 °C.

### BLI

All the BLI measurements were conducted using a Fortebio (Sartorius) K2 Octet using fiber optic biosensors. Anti–penta-His biosensors were loaded using purified WDR44, which had 10x His tag on the N terminus. Fiber tips were preincubated in the BLI buffer (20 mM Hepes [pH 7.5], 150 mM NaCl, 0.01%, 1 mM MgCl_2_, bovine serum albumin, and 0.002% Tween-20) for 10 min before experiments began. The sequence of steps in each assay was regeneration, custom, loading, baseline, association, and dissociation. Every experiment was done at 30 °C and with shaking at 1000 rpm. Technical replicates were performed by using the same fiber tip and repeating the steps outlined previously. Regeneration was done by dipping the tips back and forth six times (5 s per each) between glycine (pH 1.5) and BLI buffer. BLI buffer was used for the custom, baseline, and dissociation steps; all steps were performed in the same well for a given sample. His-Strep-WDR44 constructs were diluted in BLI buffer to 25 nM and were loaded onto the anti–penta-His fiber tips. Rab11 was also diluted in BLI buffer to 500 or 75 nM and added to the appropriate association wells. BLI experiments were done using two or three technical replicates. Means ± SD were used to present values. Statistical analysis between conditions was performed using an unpaired *t* test analyzing mutant responses to wildtype responses measured simultaneously during each assay. The concentration of a given mutant was compared with the same concentration for wildtype binding. The following legends are used for statistical significance: ∗*p* < 0.05 and ∗∗*p* < 0.01.

### HDX-MS sample preparation

HDX reactions comparing phosphorylated and unphosphorylated WDR44, phosphorylated and unphosphorylated WDR44 Rab11 complex, and Rab11 were carried out in 20 μl reactions. Reactions contained either 1.25 μM (25 pmol) of phosphorylated or unphosphorylated WDR 44 or equivalent volume of WDR44 buffer (20 mM Hepes [pH 7.0], 150 mM NaCl, 1 mM TCEP, and 2 mM MgCl_2_) and 2.5 μM (50 pmol) Rab11 or equivalent volume of Rab11 buffer (20 mM Hepes [pH 7.0], 150 mM NaCl, 1 mM TCEP, and 2 mM MgCl_2_). Exchange reactions were initiated by the addition of 17.25 μl of deuterium oxide (D2O) buffer (10 mM Hepes [pH 7.5], 50 mM NaCl, 97.09% D2O [v/v]) to 2.75 μl of protein mixture (final D2O concentration of 85.2%) and proceeded for 3, 30, 300, or 3000 s at room temperature or 0.3 s (3 s on ice). HDX reactions comparing apo-Rab11, the Rab11–WDR44 complex, and the Rab11–Fip3 complex were carried out in 40 μl reactions. Reactions contained 0.5 μM (20 pmol) of Rab11, 0.75 μM (30 pmol) of WDR44 or WDR44 buffer (100 mM NaCl, 20 mM Hepes [pH 7.5], and 1 mM TCEP), and 0.75 μM (30 pmol) of Fip3 or Fip3 buffer (100 mM NaCl, 20 mM Hepes [pH 7.5], and 1 mM TCEP). Exchange reactions were initiated by the addition of 32 μl of D2O buffer (20 mM Hepes [pH 7.5], 100 mM NaCl, 94.33% D2O [v/v]) to 8 μl of protein mixture (final D2O concentration of 75.5%), and proceeded for 3, 30, 300, or 3000 s at room temperature.

All exchange reactions were quenched with ice-cold acidic quench buffer, resulting in a final concentration of 0.6 M guanidine–HCl and 0.9% formic acid post quench. All conditions and time points were created and run in triplicate. Samples were flash frozen immediately after quenching and stored at −80 °C until injected onto the ultraperformance liquid chromatography (UPLC) system for proteolytic cleavage, peptide separation, and injection onto a QTOF for mass analysis, described later.

### HDX-MS data analysis: protein digestion and MS/MS data collection

Protein samples were rapidly thawed and injected onto an integrated fluidics system containing a HDx-3 PAL liquid handling robot and climate-controlled (2 °C) chromatography system (LEAP Technologies), a Dionex Ultimate 3000 UHPLC system, as well as an Impact HD QTOF Mass spectrometer (Bruker). The full details of the automated LC system are described (41). Samples comparing phosphorylated and unphosphorylated WDR44 bound to Rab11 were run over one immobilized pepsin column (Trajan; ProDx protease column, 2.1 mm × 30 mm PDX.PP01-F32) at 200 μl/min for 3 min at 8 °C. Samples comparing apo-Rab11, the Rab11–WDR44 complex, and the Rab11–Fip3 complex were run over a different immobilized pepsin column (Waters Enzymate BEH pepsin column, 2.1 mm × 30 mm PDX 186007233). Peptides were collected and desalted on a C18 trap column (Acquity UPLC BEH C18 1.7 mm column (2.1 × 5 mm); Waters 186003975). The trap was subsequently eluted in line with an ACQUITY 1.7 μm particle, 100 × 1 mm^2^ C18 UPLC column (Waters), using a gradient of 3 to 35% B (buffer A: 0.1% formic acid; buffer B: 100% acetonitrile) over 11 min immediately followed by a gradient of 35 to 80% over 5 min. MS experiments acquired over a mass range from 150 to 2200 *m/z* using an electrospray ionization source operated at a temperature of 200 °C and a spray voltage of 4.5 kV. The resulting MS/MS datasets were analyzed using PEAKS7 (PEAKS), and a false discovery rate was set at 1% using a database of purified proteins and known contaminants (42).

### Peptide identification

Peptides were identified from the nondeuterated samples of WDR44 or Rab11 using data-dependent acquisition following tandem MS/MS experiments (0.5 s precursor scan from 150 to 2000 *m/z*; 12 0.25 s fragment scans from 150 to 2000 *m/z*). MS/MS datasets were analyzed using PEAKS7, and peptide identification was carried out by using a false discovery–based approach, with a threshold set to 0.1% using a database of purified proteins and known contaminants. The search parameters were set with a precursor tolerance of 20 ppm, fragment mass error of 0.2 Da, charge states from 1 to 8, and variable phosphorylation on Ser, Thr, and Tyr, leading to a selection criterion of peptides that had a −10logP score of 39.8 and 34.7 for WDR44 and Rab11, respectively, and a −10logP score of 25.7 for apo-Rab11.

### Mass analysis of peptide centroids and measurement of deuterium incorporation

HDExaminer Software (Sierra Analytics) was used to automatically calculate the level of deuterium incorporation into each peptide. All peptides were manually inspected for correct charge state and the presence of overlapping peptides. Deuteration levels were calculated using the centroid of the experimental isotope clusters. Results are presented as relative levels of deuterium incorporation, and the only control for back exchange was the level of deuterium present in the buffer. Differences in exchange in a peptide were considered significant if they met all three of the following criteria: >5% change in exchange, >0.5 Da difference in exchange, and a *p* value <0.01 using a two-tailed Student's *t* test. To allow for visualization of differences across all peptides, we utilized number of deuteron difference (#D) plots (Figs. 2*F*, 4*E*, and 6*C*), with plots showing the total difference in deuterium incorporation over the entire H/D exchange time course, with each point indicating a single peptide. %D plots were used to map the deuterium incorporation time course of individual peptides (Figs. 2*E*, 4*D*, and 6*C*). The entire HDX-MS dataset with all values and statistics is provided in the source data. Samples were only compared within a single experiment and never compared with experiments completed at a different time with a different final D2O level. The data analysis statistics for all HDX-MS experiments are provided in Tables S1–S3 according to the guidelines (43). HDX-MS proteomics data generated in this study have been deposited to the ProteomeXchange Consortium via the PRIDE partner repository (44) with the dataset identifier PXD035741.

## Data availability

All underlying raw data for this article are provided in the attached excel file, along with all SDS-gel images for all purified proteins. The MS proteomics data have been deposited to the ProteomeXchange Consortium *via* the PRIDE partner repository with the dataset identifier PXD035741.

## Supporting information

This article contains supporting information.

## Conflict of interest

J. E. B. reports personal fees from Scorpion Therapeutics and Olema Oncology and research grants from Novartis. All other authors declare that they have no conflicts of interest with the contents of this article.

## References

[1] Zhen Y., Stenmark H. (2015). Cellular functions of Rab GTPases at a glance. J. Cell. Sci..

[2] Müller M.P., Goody R.S. (2017). Molecular control of Rab activity by GEFs, GAPs and GDI. Small GTPases.

[3] Pylypenko O., Hammich H., Yu I.-M., Houdusse A. (2017). Rab GTPases and their interacting protein partners: structural insights into Rab functional diversity. Small GTPases.

[4] Wandinger-Ness A., Zerial M. (2014). Rab proteins and the compartmentalization of the endosomal system. Cold Spring Harb. Perspect. Biol..

[5] Welz T., Wellbourne-Wood J., Kerkhoff E. (2014). Orchestration of cell surface proteins by Rab11. Trends Cell Biol..

[6] Knödler A., Feng S., Zhang J., Zhang X., Das A., Peränen J. (2010). Coordination of Rab8 and Rab11 in primary ciliogenesis. Proc. Natl. Acad. Sci. U. S. A..

[7] Eathiraj S., Pan X., Ritacco C., Lambright D.G. (2005). Structural basis of family-wide Rab GTPase recognition by rabenosyn-5. Nature.

[8] Wittinghofer A., Vetter I. (2011). Structure-function relationships of the G domain, a canonical switch motif. Annu. Rev. Biochem..

[9] Horgan C.P., McCaffrey M.W. (2009). The dynamic Rab11-FIPs. Biochem. Soc. Trans..

[10] Eathiraj S., Mishra A., Prekeris R., Lambright D.G. (2006). Structural basis for Rab11-mediated recruitment of FIP3 to recycling endosomes. J. Mol. Biol..

[11] Shiba T., Koga H., Shin H.W., Kawasaki M., Kato R., Nakayama K. (2006). Structural basis for Rab11-dependent membrane recruitment of a family of Rab11-interacting protein 3 (FIP3)/Arfophilin-1. Proc. Natl. Acad. Sci. U. S. A..

[12] Jagoe W.N., Lindsay A.J., Read R.J., McCoy A.J., McCaffrey M.W., Khan A.R. (2006). Crystal structure of rab11 in complex with rab11 family interacting protein 2. Structure.

[13] Pylypenko O., Attanda W., Gauquelin C., Lahmani M., Coulibaly D., Baron B. (2013). Structural basis of myosin V Rab GTPase-dependent cargo recognition. Proc. Natl. Acad. Sci. U. S. A..

[14] Wu S., Mehta S.Q., Pichaud F., Bellen H.J., Quiocho F.A. (2005). Sec15 interacts with Rab11 *via* a novel domain and affects Rab11 localization *in vivo*. Nat. Struct. Mol. Biol..

[15] Vetter M., Stehle R., Basquin C., Lorentzen E. (2015). Structure of Rab11-FIP3-Rabin8 reveals simultaneous binding of FIP3 and Rabin8 effectors to Rab11. Nat. Struct. Mol. Biol..

[16] Burke J.E., Inglis A.J., Perisic O., Masson G.R., McLaughlin S.H., Rutaganira F. (2014). Structures of PI4KIIIβ complexes show simultaneous recruitment of Rab11 and its effectors. Science.

[17] Westlake C.J., Inglis A.J., Perisic O., Masson G.R., McLaughlin S.H., Rutaganira F. (2011). Primary cilia membrane assembly is initiated by Rab11 and transport protein particle II (TRAPPII) complex-dependent trafficking of Rabin8 to the centrosome. Proc. Natl. Acad. Sci. U. S. A..

[18] Mammoto A., Ohtsuka T., Hotta I., Sasaki T., Takai Y. (1999). Rab11BP/Rabphilin-11, a downstream target of rab11 small G protein implicated in vesicle recycling. J. Biol. Chem..

[19] Zeng J., Ren M., Gravotta D., De Lemos-Chiarandini C., Lui M., Erdjument-Bromage H. (1999). Identification of a putative effector protein for rab11 that participates in transferrin recycling. Proc. Natl. Acad. Sci. U. S. A..

[20] Lucken-Ardjomande Häsler S., Vallis Y., Pasche M., McMahon H.T. (2020). GRAF2, WDR44, and MICAL1 mediate Rab8/10/11-dependent export of E-cadherin, MMP14, and CFTR ΔF508. J. Cell Biol..

[21] Walia V., Cuenca A., Vetter M., Insinna C., Perera S., Lu Q. (2019). Akt regulates a rab11-effector switch required for ciliogenesis. Dev. Cell.

[22] Malik N., Nirujogi R.S., Peltier J., Macartney T., Wightman M., Prescott A.R. (2019). Phosphoproteomics reveals that the hVPS34 regulated SGK3 kinase specifically phosphorylates endosomal proteins including Syntaxin-7, Syntaxin-12, RFIP4 and WDR44. Biochem. J..

[23] Varadi M., Anyango S., Deshpande M., Nair S., Natassia C., Yordanova G. (2022). AlphaFold protein structure database: massively expanding the structural coverage of protein-sequence space with high-accuracy models. Nucl. Acids Res..

[24] Jumper J., Evans R., Pritzel A., Green T., Figurnov M., Ronneberger O. (2021). Highly accurate protein structure prediction with AlphaFold. Nature.

[25] Mirdita M., Schütze K., Moriwaki Y., Heo L., Ovchinnikov S., Steinegger M. (2022). ColabFold: making protein folding accessible to all. Nat. Met..

[26] Pasqualato S., Senic-Matuglia F., Renault L., Goud B., Salamero J., Cherfils J. (2004). The structural GDP/GTP cycle of Rab11 reveals a novel interface involved in the dynamics of recycling endosomes. J. Biol. Chem..

[27] Merithew E., Hatherly S., Dumas J.J., Lawe D.C., Heller-Harrison R., Lambright D.G. (2001). Structural plasticity of an invariant hydrophobic triad in the switch regions of Rab GTPases is a determinant of effector recognition. J. Biol. Chem..

[28] Skinner J.J., Lim W.K., Bédard S., Black B.E., Englander S.W. (2012). Protein dynamics viewed by hydrogen exchange. Protein Sci..

[29] Masson G.R., Jenkins M.L., Burke J.E. (2017). An overview of hydrogen deuterium exchange mass spectrometry (HDX-MS) in drug discovery. Expert Opin. Drug Discov..

[30] Ostermeier C., Brunger A.T. (1999). Structural basis of rab effector specificity: Crystal structure of the small G protein Rab3A complexed with the effector domain of rabphilin-3A. Cell.

[31] Luo L., Wall A.A., Yeo J.C., Condon N.D., Norwood S.J., Schoenwaelder S. (2014). Rab8a interacts directly with PI3Kγ to modulate TLR4-driven PI3K and mTOR signalling. Nat. Commun..

[32] Waschbüsch D., Khan A.R. (2020). Phosphorylation of Rab GTPases in the regulation of membrane trafficking. Traffic.

[33] Steger M., Tonelli F., Ito G., Davies P., Trost M., Vetter M. (2016). Phosphoproteomics reveals that Parkinson’s disease kinase LRRK2 regulates a subset of Rab GTPases. Elife.

[34] Steger M., Diez F., Dhekne H.S., Lis P., Nirujogi R.S., Karayel O. (2017). Systematic proteomic analysis of LRRK2-mediated Rab GTPase phosphorylation establishes a connection to ciliogenesis. Elife.

[35] Waschbüsch D., Purlyte E., Pal P., McGrath E., Alessi D.R., Khan A.R. (2020). Structural basis for Rab8a recruitment of RILPL2 *via* LRRK2 phosphorylation of switch 2. Structure.

[36] Jenkins M.L., Harris N.J., Dalwadi U., Fleming K.D., Ziemianowicz D.S., Rafiei A. (2020). The substrate specificity of the human TRAPPII complex’s Rab-guanine nucleotide exchange factor activity. Commun. Biol..

[37] Harris N.J., Jenkins M.L., Dalwadi U., Fleming K.D., Nam S.E., Parson M.A.H. (2021). Biochemical insight into novel rab-GEF activity of the mammalian TRAPPIII complex. J. Mol. Biol..

[38] Gouet P., Robert X., Courcelle E. (2003). ESPript/ENDscript: extracting and rendering sequence and 3D information from atomic structures of proteins. Nucl. Acids Res..

[39] Evans R., O’Neill M., Pritzel A., Antropova N., Senior A., Green T. (2021). Protein complex prediction with AlphaFold-Multimer. bioRxiv.

[40] Pokorny D., Truebestein L., Fleming K.D., Burke J.E., Leonard T.A. (2021). *In vitro* reconstitution of Sgk3 activation by phosphatidylinositol 3-phosphate. J. Biol. Chem..

[41] Stariha J.T.B., Hoffmann R.M., Hamelin D.J., Burke J.E. (2021). Probing protein-membrane interactions and dynamics using hydrogen-deuterium exchange mass spectrometry (HDX-MS). Met. Mol. Biol..

[42] Dobbs J.M., Jenkins M.L., Burke J.E. (2020). Escherichia coli and Sf9 contaminant databases to increase efficiency of tandem mass spectrometry peptide identification in structural mass spectrometry experiments. J. Am. Soc. Mass Spectrom..

[43] Masson G.R., Burke J.E., Ahn N.G., Anand G.S., Borchers C., Brier S. (2019). Recommendations for performing, interpreting and reporting hydrogen deuterium exchange mass spectrometry (HDX-MS) experiments. Nat. Met..

[44] Perez-Riverol Y., Bai J., Bandla C., García-Seisdedos D., Hewapathirana S., Kamatchinathan S. (2022). The PRIDE database resources in 2022: a hub for mass spectrometry-based proteomics evidences. Nucl. Acids Res..

