# Supplementary material for: Molecular basis for the recruitment of the Rab effector protein WDR44 by the GTPase Rab11
Source: J Biol Chem. 2022 Dec 1;299(1):102764. doi: 10.1016/j.jbc.2022.102764 (PMC9808001; doi:10.1016/j.jbc.2022.102764)
Supplement: Supplemental figures [file mmc1.pdf]

Supplemental Figures and Figure Legends for

Molecular basis for the recruitment of the Rab effector protein WDR44 by the GTPase Rab11

Matthew C Thibodeau[1*], Noah J Harris[1*], Meredith L Jenkins[1], Matthew AH Parson[1], John Evans[1], Mackenzie Scott[1], Alexandria L Shaw[1,2], Daniel Pokorný[3], Thomas A Leonard[3], John E Burke[1,2#]

[1]Department of Biochemistry and Microbiology, University of Victoria, Victoria, British Columbia, V8W 2Y2, Canada

[2]Department of Biochemistry and Molecular Biology, The University of British Columbia, Vancouver, British Columbia V6T 1Z3, Canada

[3]Max Perutz Labs, Department of Structural and Computational Biology, University of Vienna, 1030 Vienna, Austria

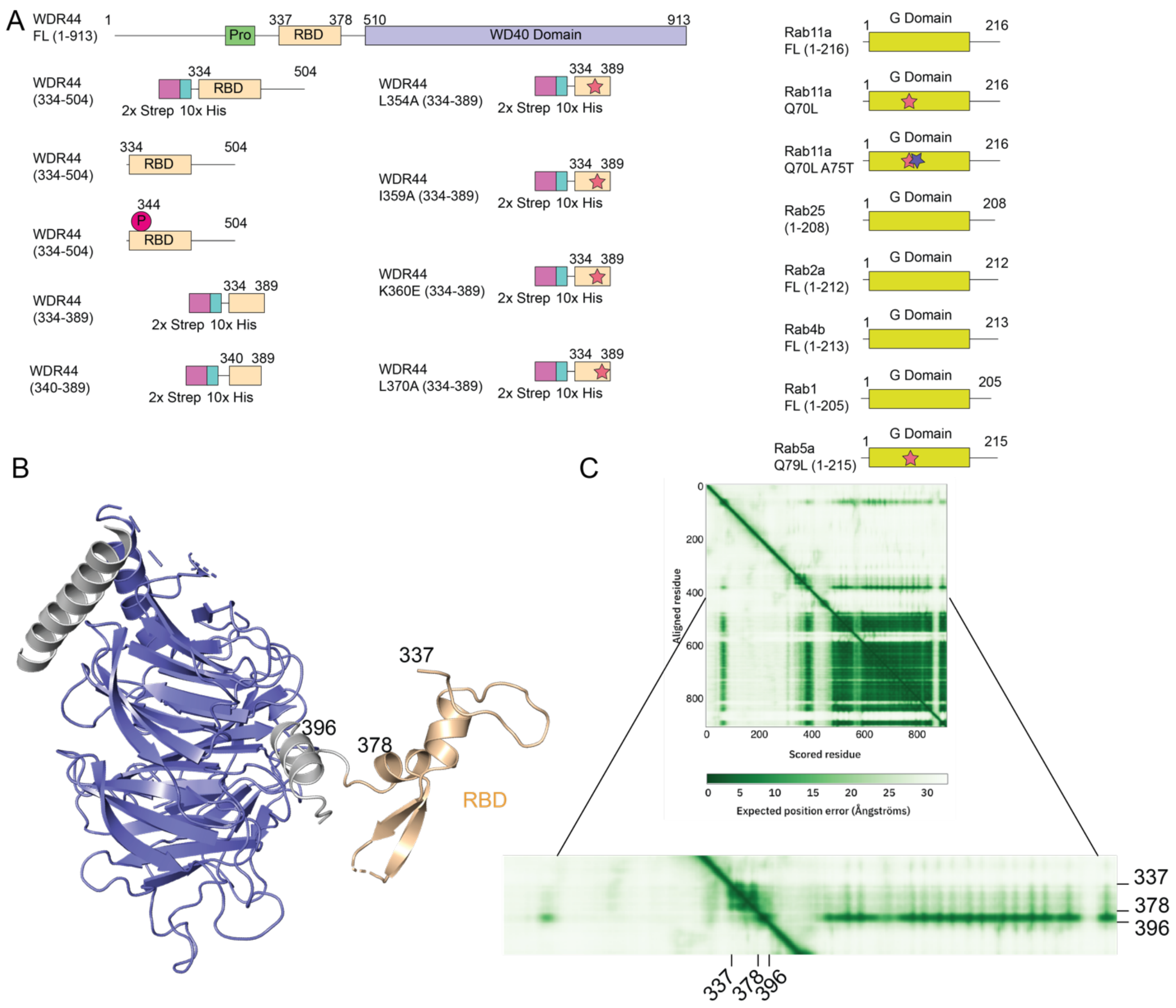

Figure S1. Protein constructs used and predicted alignment error of WDR44.

A. Purified constructs of WDR44 and Rab GTPases used in experiments with full length WDR44 for reference.

B. AlphaFold structure of WDR44 with residues pLDDT < 50 not shown. The helix spanning residues 383-392 is shown in gray, with the RBD domain shown in light brown.

C. Predicted aligned error of WDR44 for each residue relative to all other residues of WDR44. The colour at (x, y) corresponds to the expected distance error in residue x's position (Angstroms), when the prediction are aligned on residue y (more information can be found at https://alphafold.ebi.ac.uk/) [30,31]. The PAE of the region spanning the RBD is zoomed in on, showing limited correlation between the RBD and the WD40 domain, with a single helix C-terminal to the RBD (383-392) forming a stable interface with the WD40 domain.

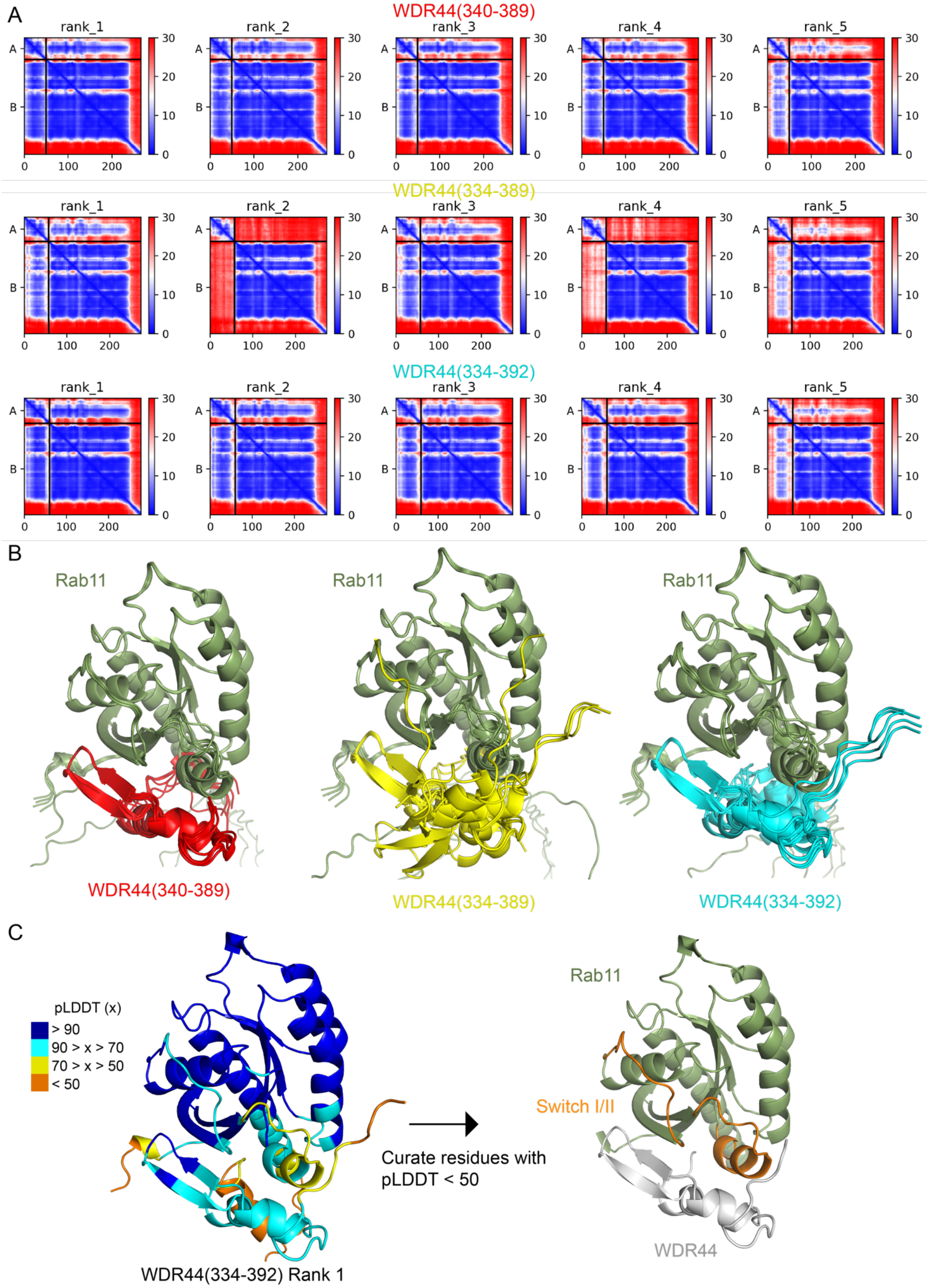
A
WDR44(340-389)
rank_1
rank_2
rank_3
rank_4
rank_5
WDR44(334-389)
WDR44(334-392)
B
Rab11
WDR44(340-389)
WDR44(334-389)
WDR44(334-392)
C
pLDDT (x)
> 90
90 > x > 70
70 > x > 50
< 50
WDR44(334-392) Rank 1
Curate residues with
pLDDT < 50
Rab11
Switch I/II
WDR44

Figure S2. Validation of Alphafold modelling of the WDR44-Rab11 complex.

A. Predicted aligned error (PAE) for Alphafold2 search of the full length Rab11a Q70L with various WDR44 constructs. The sequences of the searches are indicated, with corresponding structures below. The colours indicate the predicted aligned error, and are coloured according to the legend. Note that the PAE plot is not an inter-residue distance map or a contact map. Instead, the red-blue colour indicates expected distance error. The colour at (x, y) corresponds to the expected distance error in residue x's position (Angstroms), when the prediction are aligned on residue y (more information can be found at https://alphafold.ebi.ac.uk/) [30,31].

B. Overlaid structures of Alphafold model ranks 1-5 for each WDR44 construct. Structural models correspond to the PAE for each construct from the panel above.

C. Alphafold2 model of WDR44 (334-392) shown with the per-residue confidence metric predicted local-distance difference test (pLDDT) coloured according to the legend. The pLDDT score varies from 0 to 100, and is an estimate of how well the prediction would agree with an experimental structure based on the local distance difference test Cα [30].

A

B

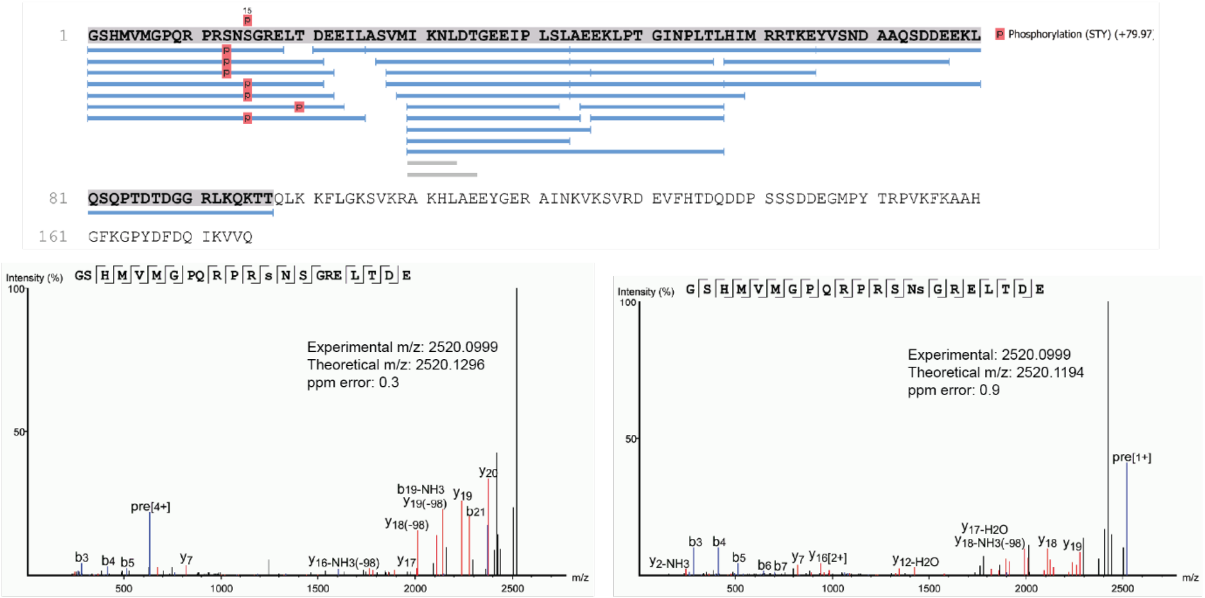

Figure S3. Phosphorylated WDR MS/MS spectra

A. MS/MS of SGK3 phosphorylated WDR44 (334-504) sequence showing peptides identified, as well as putative phosphorylation sites. It should be noted that fragmentation was carried out with CID, leading to loss of phosphorylation in fragments, causing MS identification of the exact phosphorylation site to be ambiguous.

B. MS/MS spectra of two peptides from panel **A** covering the phosphorylation site. Identified b/y fragments are annotated on the peptide sequence, with fragments with -98 denote loss of phosphate annotated on the spectra. The theoretical and experimental masses for the precursor ions are annotated, with ppm error included.

Supplementary Table S1. HDX Data Summary apo Rab11, Rab11-phosphorylated WDR44, Rab11-unphosphorylated WDR44.

Protein Data Set	Rab11 Apo	Rab11 Phosphorylated Complex	Rab11 Unphosphorylated Complex
HDX reaction details	$\%D_2O$= 85.25% pH(read)= 7.5 Temp= 18°C	$\%D_2O$= 85.25% pH(read)= 7.5 Temp= 18°C	$\%D_2O$= 85.25% pH(read)= 7.5 Temp= 18°C
HDX time course	0.3s (3s on ice), 3s, 30s, 300s, 3000s	0.3s (3s on ice), 3s, 30s, 300s, 3000s	0.3s (3s on ice), 3s, 30s, 300s, 3000s
HDX controls	N/A	N/A	N/A
Back-exchange	Corrected based on $\%D_2O$	Corrected based on $\%D_2O$	Corrected based on $\%D_2O$
Number of peptides	87	87	87
Sequence coverage	92.9%	92.9%	92.9%
Average peptide length / Redundancy	Length = 16.9 Redundancy = 6.5	Length = 16.9 Redundancy = 6.5	Length = 16.9 Redundancy = 6.5
Replicates	3	3 (2 for 30s timepoint)	3
Repeatability	Average StDev = 0.7%	Average StDev = 0.5%	Average StDev = 0.6%
Significant differences in HDX	>5% and >0.5 Da and unpaired t-test <0.01	>5% and >0.5 Da and unpaired t-test <0.01	>5% and >0.5 Da and unpaired t-test <0.01

Supplementary Table S2 HDX Data Summary apo WDR44, phosphorylated WDR44, phosphorylated WDR44-Rab11, unphosphorylated WDR44-Rab11.

Protein Data Set	WDR44 Phosphorylated Apo	WDR44 Unphosphorylated Apo	WDR44 Phosphorylated Complex	WDR44 Unphosphorylated Complex
HDX reaction details	$\%D_2O$= 85.25% pH(read)= 7.5 Temp= 18°C	$\%D_2O$= 85.25% pH(read)= 7.5 Temp= 18°C	$\%D_2O$= 85.25% pH(read)= 7.5 Temp= 18°C	$\%D_2O$= 85.25% pH(read)= 7.5 Temp= 18°C
HDX time course	0.3s (3s on ice), 3s, 30s, 300s, 3000s	0.3s (3s on ice), 3s, 30s, 300s, 3000s	0.3s (3s on ice), 3s, 30s, 300s, 3000s	0.3s (3s on ice), 3s, 30s, 300s, 3000s
HDX controls	N/A	N/A	N/A	N/A
Back-exchange	Corrected based on $\%D_2O$	Corrected based on $\%D_2O$	Corrected based on $\%D_2O$	Corrected based on $\%D_2O$
Number of peptides	34	34	34	34
Sequence coverage	64%	64%	64%	64%
Average peptide length / Redundancy	Length = 17.9 Redundancy = 2.7	Length = 17.9 Redundancy = 2.7	Length = 17.9 Redundancy = 2.7	Length = 17.9 Redundancy = 2.7
Replicates	3 (2 for 3s timepoint)	3 (2 for 30s timepoint)	3	3
Repeatability	Average StDev = 1.2%	Average StDev = 1.1%	Average StDev = 1.2%	Average StDev = 1.4%
Significant differences in HDX	>5% and >0.5 Da and unpaired t-test <0.01	>5% and >0.5 Da and unpaired t-test <0.01	>5% and >0.5 Da and unpaired t-test <0.01	>5% and >0.5 Da and unpaired t-test <0.01

Supplementary Table S3. HDX Data Summary apo Rab11, WDR44-Rab11, and FIP3-Rab11.

Protein Data Set	Rab11 Apo	Rab11 WDR44 Complex	Rab11 Fip Complex
HDX reaction details	%D_2O= 75.5%<br>pH(read)= 7.5<br>Temp= 18°C	%D_2O= 75.5%<br>pH(read)= 7.5<br>Temp= 18°C	%D_2O= 75.5%<br>pH(read)= 7.5<br>Temp= 18°C
HDX time course	3s, 30s, 300s	3s, 30s, 300s	3s, 30s, 300s
HDX controls	N/A	N/A	N/A
Back-exchange	Corrected based on %D_2O	Corrected based on %D_2O	Corrected based on %D_2O
Number of peptides	68	68	68
Sequence coverage	98.2%	98.2%	98.2%
Average peptide length / Redundancy	Length = 17.3<br>Redundancy = 5.4	Length = 17.3<br>Redundancy = 5.4	Length = 17.3<br>Redundancy = 5.4
Replicates	3	3	3
Repeatability	Average StDev = 0.7%	Average StDev = 0.4%	Average StDev = 0.4%
Significant differences in HDX	>5% and >0.5 Da and unpaired t-test <0.01	>5% and >0.5 Da and unpaired t-test <0.01	>5% and >0.5 Da and unpaired t-test <0.01
